# The actual 5-year survivors of pancreatic ductal adenocarcinoma based on real-world data

**DOI:** 10.1038/s41598-020-73525-y

**Published:** 2020-10-02

**Authors:** Axel Bengtsson, Roland Andersson, Daniel Ansari

**Affiliations:** Department of Surgery, Clinical Sciences Lund, Lund University, Skåne University Hospital, 221 85 Lund, Sweden

**Keywords:** Gastroenterology, Oncology

## Abstract

Survival data for pancreatic cancer are usually based on actuarial calculations and actual long-term survival rates are rarely reported. Here we use population-level data from the Surveillance, Epidemiology, and End Results program for patients with microscopically confirmed pancreatic ductal adenocarcinoma diagnosed from 1975 to 2011. A total of 84,275 patients with at least 5 years of follow-up were evaluated (follow-up cutoff date: December 31, 2016). Actual 5-year survival for pancreatic cancer increased from 0.9% in 1975 to 4.2% in 2011 in patients of all stages (p < 0.001), while in surgically resected patients, it rose from 1.5% to 17.4% (p < 0.001). In non-resected patients, the actual 5-year survival remained unchanged over the same time period (0.8% vs 0.9%; p = 0.121). Multivariable analysis of surgically resected patients diagnosed in the recent time era (2004–2011) showed that age, gender, grade, tumour size, TNM-stage and chemotherapy were significant independent predictors of actual 5-year survival, while age, grade and TNM-stage were significant independent predictors in non-resected patients. However, unfavourable clinicopathological factors did not preclude long-term survival. Collectively, our findings indicate that actual 5-year survival for pancreatic cancer is still below 5% despite improvement of survival for the subset of patients undergoing surgical resection.

## Introduction

Pancreatic cancer carries the lowest survival rate of all major organ cancers and is the third leading contributor to cancer mortality in the United States^1^. Following diagnosis, survival typically ranges from 4 to 6 months^2^. Although 5-year survival rates of up to 30–58% in resected pancreatic cancer patients have been reported, the data for actual 5-year survival are more modest^3^. Several series have failed to present any 5-year survivors and there are those that suggest that the overall actual survival rate is below 0.3% when all stages are combined^4,5^.

Prognostic factors for short-term survival in pancreatic cancer are well reported. However, factors predicting long-term survival are less understood. Clinical and pathological features predictive of actuarial 5-year survival may not reflect the factors specific to actual long-term survivors (LTS)^6,7^. This is due to the high early disease-related mortality seen in pancreatic cancer^8^ in addition to the inflated survival present in actuarial data when loss of follow-up is present in the patient material^3^. Therefore, predictors of long-term survival in population-based data, where exclusion of patients lost to follow up is a prerequisite for the comparison of variables among subgroups, must be based on actual rather than actuarial data.

The disparities in patient characteristics between clinical trials and registries presenting real world data (RWD) are reflected in the survival of pancreatic cancer, with clinical trial patients having markedly improved survival compared to national database populations^9^. Thus, when describing characteristics of patient subgroups such as LTS in terms of predictors, contemporary population-based RWD are better able to make generalisations pertaining to the real-world effect of modern treatment strategies^10,11^.

Nearly all previous studies of actual LTS (≥ 5 years) in pancreatic cancer have been single-institution series and describe a limited number of patients. Here, we use data from the National Cancer Institute’s Surveillance, Epidemiology, and End Results (SEER) program in the era following the publication of the final results of the ESPAC 1-trial^12^ of adjuvant chemotherapy in pancreatic cancer. The aim of the present study was to analyse trends in actual long-term survival of pancreatic cancer and to identify predictors of long-term survival in the recent time era.

## Results

### Patient characteristics

Data were obtained from 181,392 patients with pancreatic cancer registered in the SEER database between 1975 and 2011. Some 74,367 patients lacking histological or cytological confirmation of the tumour and 22,750 patients with no information on cause of death or vital status or incomplete follow-up time were excluded. The final study population comprised 84,275 patients with pancreatic ductal adenocarcinoma, out of whom 38,709 patients were diagnosed between 2004 and 2011. Some 2440 patients (2.9%) of our total study population had a survival exceeding 5 years.

The characteristics of the study population are described in Table 1. Median age at diagnosis was 68 years and 48.7% were female. Some 7.1% of patients had a localized tumour, 31.2% had regional spread, 56.0% had distant disease and 5.7% were unstaged. Some 18.5% of patients underwent surgical resection, 46.1% received chemotherapy and 20.7% received radiation.

**Table 1:** Characteristics of 84275 patients with microscopically confirmed pancreatic ductal adenocarcinoma diagnosed from 1975 to 2011.

Variables	Overall (N = 84,275)
Age (years)	68 (59–76)
Female gender	41,065 (48.7%)
**SEER historic stage A**^a^	
Localized	5967 (7.1%)
Regional	26,318 (31.2%)
Distant	47,228 (56.0%)
Unstaged	4762 (5.7%)
**Surgical resection**	
Yes	15,618 (18.5%)
No	66,269 (78.6%)
Unknown	2388 (2.8%)
**Chemotherapy**	
Yes	38,829 (46.1%)
No^b^	45,446 (53.9%)
**Radiation**	
Yes	17,428 (20.7%)
No^c^	66,057 (78.4%)
Unknown	790 (0.9%)

^a^SEER historic stage A is presented here because AJCC stage was not available for the older time period.

^b^No evidence of chemotherapy was found in the medical records examined.

^c^No evidence of radiation was found in the medical records examined.

Demographic and clinical data from the LTS were compared with those of the STS for the recent time era (2004–2011), as shown in Table 2. LTS were significantly younger at diagnosis and were more often female. Tumours of LTS were more likely to be smaller in size. However, unfavourable clinicopathological factors did not preclude long-term survival. The majority of LTS received surgical resection (82.8%), while only 17.2% underwent resection among STS. Chemotherapy and radiation were also administered more frequently in LTS.

**Table 2:** Comparison of long-term and short-term survivors of pancreatic ductal adenocarcinoma who were diagnosed in the recent time era (2004 to 2011).

Variables	N	Short-term survivors (< 5 years) N = 37,235	Long-term survivors (≥ 5 years) N = 1473	p value
Age (years)	38,708	67 (59–76)	65 (57–72)	< 0.001
Female gender	38,708	18,014 (48.4%)	768 (52.1%)	0.005
**Tumour location**	38,708			< 0.001
Head		19,083 (51.3%)	1023 (69.5%)	
Body		4473 (12.0%)	104 (7.1%)	
Tail		4619 (12.4%)	146 (9.9%)	
Other^a^		9060 (24.3%)	200 (13.6%)	
**Grade**	15,061			< 0.001
Well differentiated		1440 (10.3%)	215 (18.8%)	
Moderately differentiated		6079 (43.7%)	601 (52.5%)	
Poorly differentiated/anaplastic		6397 (46.0%)	329 (28.7%)	
**Tumour size (cm)**	28,788	3.8 (3–5)	2.8 (2–3.9)	< 0.001
**AJCC stage 7th edition**	35,943			< 0.001
Stage I		1690 (4.9%)	328 (23.7%)	
Stage II		9320 (27.0%)	876 (63.2%)	
Stage III		3708 (10.7%)	72 (5.2%)	
Stage IV		19,840 (57.4%)	109 (7.9%)	
**T stage**	29,949			< 0.001
T1		882 (3.1%)	185 (13.4%)	
T2		6157 (21.5%)	272 (19.8%)	
T3		13,902 (48.7%)	828 (60.2%)	
T4		7632 (26.7%)	91 (6.6%)	
**N stage**	30,893			0.333
N0		16,987 (57.6%)	821 (58.9%)	
N1		12,512 (42.4%)	573 (41.1%)	
**M stage**	37,188			< 0.001
M0		15,919 (44.5%)	1320 (92.4%)	
M1		19,840 (55.5%)	109 (7.6%)	
Surgical resection	38,336	6358 (17.2%)	1206 (82.8%)	< 0.001
Chemotherapy^b^	38,708	20,034 (53.8%)	1037 (70.4%)	< 0.001
Radiation^c^	38,458	7095 (19.2%)	615 (42.2%)	< 0.001

*N* number of non-missing values.

^a^Other sites include C25.3, pancreatic duct; C25.7, other specified parts of pancreas; C25.8, overlapping lesion of pancreas; C25.9, pancreas, not otherwise specified.

^b^Chemotherapy data classified as “yes” or “no/unknown – no evidence of chemotherapy was found in the medical records examined”.

^c^Radiation data classified as “yes” or “no/unknown – no evidence of radiation was found in the medical records examined”.

### Univariable and multivariable analysis of factors associated with LTS

We examined factors associated with LTS in both univariable and multivariable logistic regression (Table 3). When considering patients of all stages, age, gender, T-stage, M-stage, tumour size, histological grade, surgical resection and chemotherapy were identified as independent predictors of LTS. Subgroup analyses were performed to determine whether the predictive factors were different between resected and non-resected patients. For surgically resected patients, age, gender, TNM-stage, tumour size, grade, and chemotherapy remained as significant independent predictors of LTS. In non-resected patients, only age, TNM-stage and grade were independent predictors of LTS.

**Table 3:** Univariable and multivariable logistic regression analyses of factors associated with long-term survivors (≥ 5 years).

	Univariable		Multivariable	
	Odds ratio (95% CI)	p value	Odds ratio (95% CI)	p value
**All patients (N = 38,708)**				
Age (years)	0.977 (0.972–0.981)	< 0.001	0.984 (0.979–0.989)	< 0.001
Female gender	1.16 (1.05–1.29)	0.005	1.13 (1.01–1.27)	0.031
**Tumour location**				
Head	1 (reference)			
Body	0.434 (0.354–0.532)	< 0.001		
Tail	0.590 (0.494–0.703)	< 0.001		
Other	0.412 (0.353–0.480)	< 0.001		
**Grade**				
Well differentiated	1 (reference)		1 (reference)	
Moderately differentiated	0.657 (0.561–0.770)	< 0.001	0.557 (0.464–0.667)	< 0.001
Poorly differentiated/anaplastic	0.319 (0.271–0.376)	< 0.001	0.382 (0.318–0.459)	< 0.001
Tumour size (cm)	0.637 (0.609–0.666)	< 0.001	0.878 (0.837–0.920)	< 0.001
**T stage**				
T1	1 (reference)		1 (reference)	
T2	0.210 (0.172–0.257)	< 0.001	0.560 (0.431–0.726)	< 0.001
T3	0.282 (0.238–0.336)	< 0.001	0.347 (0.275–0.438)	< 0.001
T4	0.061 (0.047–0.079)	< 0.001	0.247 (0.177–0.344)	< 0.001
N stage	0.972 (0.872–1.08)	0.613		
M stage	0.075 (0.062–0.091)	< 0.001	0.320 (0.256–0.400)	< 0.001
Surgical resection	23.2 (20.2–26.6)	< 0.001	10.8 (9.13–12.8)	< 0.001
Chemotherapy	2.04 (1.82–2.29)	< 0.001	1.45 (1.27–1.64)	< 0.001
Radiation	3.06 (2.74–3.40)	< 0.001		
**Surgically resected patients (N = 7564)**				
Age (years)	0.988 (0.983–0.994)	< 0.001	0.988 (0.982–0.994)	< 0.001
Female gender	1.19 (1.05–1.34)	0.007	1.18 (1.03–1.34)	0.014
**Tumour location**				
Head	1 (reference)			
Body	1.33 (1.04–1.70)	0.025		
Tail	1.26 (1.02–1.55)	0.030		
Other	0.969 (0.791–1.19)	0.762		
**Grade**				
Well differentiated	1 (reference)		1 (reference)	
Moderately differentiated	0.520 (0.425–0.635)	< 0.001	0.590 (0.474–0.734)	< 0.001
Poorly differentiated/anaplastic	0.333 (0.270–0.411)	< 0.001	0.431 (0.343–0.542)	< 0.001
Tumour size (cm)	0.753 (0.715–0.792)	< 0.001	0.857 (0.813–0.904)	< 0.001
**T stage**				
T1	1 (reference)		1 (reference)	
T2	0.439 (0.343–0.561)	< 0.001	0.680 (0.515–0.897)	0.006
T3	0.252 (0.205–0.311)	< 0.001	0.467 (0.367–0.594)	< 0.001
T4	0.092 (0.059–0.141)	< 0.001	0.176 (0.111–0.280)	< 0.001
N stage	0.348 (0.306–0.396)	< 0.001	0.384 (0.335–0.440)	< 0.001
M stage	0.189 (0.121–0.295)	< 0.001	0.236 (0.149–0.371)	< 0.001
Chemotherapy	1.46 (1.27–1.67)	< 0.001	1.68 (1.44–1.95)	< 0.001
Radiation	1.40 (1.23–1.58)	< 0.001		
**Non-resected patients (N = 30,772)**				
Age (years)	0.975 (0.965–0.985)	< 0.001	0.967 (0.957–0.977)	< 0.001
Female gender	1.02 (0.795–1.31)	0.881		
**Tumour location**				
Head	1 (reference)			
Body	0.446 (0.273–0.729)	0.001		
Tail	0.475 (0.294–0.768)	0.002		
Other	0.895 (0.673–1.19)	0.444		
**Grade**				
Well differentiated	1 (reference)		1 (reference)	
Moderately differentiated	0.491 (0.342–0.704)	< 0.001	0.540 (0.376–0.776)	0.001
Poorly differentiated/anaplastic	0.272 (0.181–0.409)	< 0.001	0.332 (0.220–0.499)	< 0.001
Tumour size (cm)	0.889 (0.814–0.970)	0.009		
**T stage**				
T1	1 (reference)		1 (reference)	
T2	0.395 (0.210–0.744)	0.004	0.416 (0.217–0.797)	0.009
T3	0.350 (0.188–0.653)	0.001	0.311 (0.164–0.591)	0.001
T4	0.382 (0.206–0.710)	0.003	0.300 (0.157–0.573)	< 0.001
N stage	0.665 (0.490–0.902)	0.009	0.701 (0.512–0.960)	0.027
M stage	0.371 (0.283–0.486)	< 0.001	0.358 (0.269–0.477)	< 0.001
Chemotherapy	1.52 (1.18–1.97)	0.001		
Radiation	1.82 (1.36–2.43)	< 0.001		

N, number of non-missing values.

### Trends in actual survival and distribution of tumour stages

**Figure 1:**
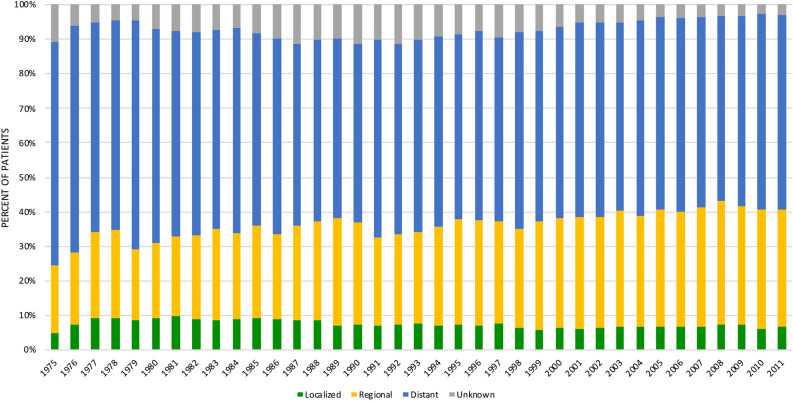
Distribution of pancreatic cancer cases by stage at diagnosis from 1975 to 2011. SEER Historic Stage A is presented to ensure uniform staging throughout the study period.

Actual 5-year survival by AJCC-stage for patients diagnosed in the recent era (2004–2011) is presented in Table 4. Actual 5-year survival was 31.7% for IA tumours, and decreased to 11.8% in stage IB patients, while stage IV tumours showed an actual 5-year survival of 0.5%. Figure 1 presents the proportions of SEER summary stage A from 1975 to 2011 in patients of all stages. The proportion of patients with localized disease was 5.4% in 1975 compared to 7.0% in 2011 (p < 0.001).

**Table 4:** Actual 5-year survival by AJCC stage for patients diagnosed in the recent time era (2004 to 2011), N = 35,628^a^.

Stage	Proportion (%)	Actual 5-year survival (%)*
**AJCC stage 7th edition**		
Stage IA	1.3	31.7
Stage IB	4.4	11.8
Stage IIA	11.5	9.0
Stage IIB	16.3	8.7
Stage III	10.6	1.9
Stage IV	56.0	0.5

^a^Number of patients with non-missing values on AJCC stage (excluding 315 patients with stage II-NOS and 2765 patients with unknown stage).

*Follow-up cutoff date: December 31, 2016.

**Figure 2:**
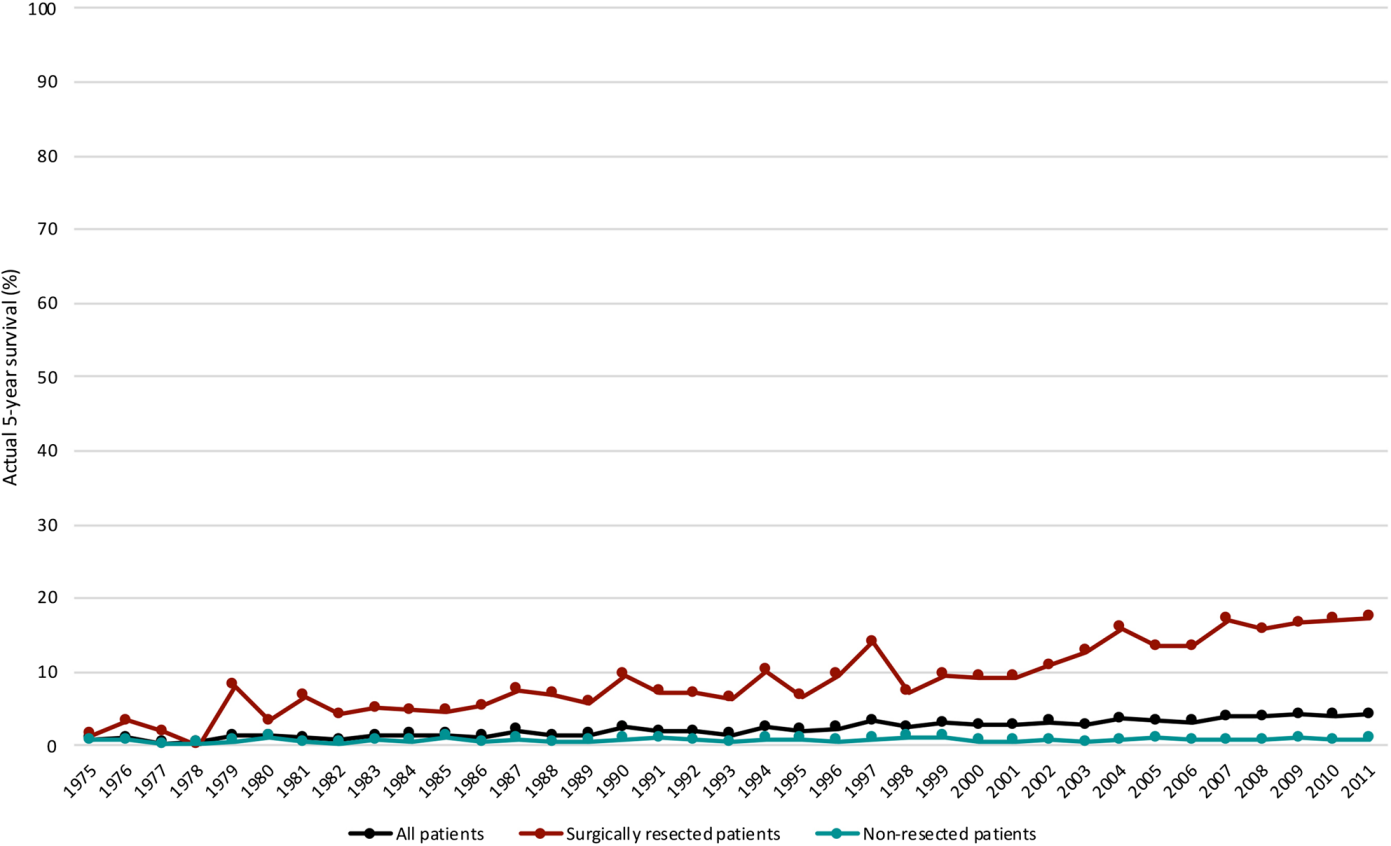
Trends in the actual 5-year survival for 84,275 patients with pancreatic ductal adenocarcinoma diagnosed from 1975 to 2011. Follow-up cutoff date: December 31, 2016.

Figure 2 depicts the trend analysis of actual 5-year survival in patients of all stages and the subgroups of surgically resected and non-resected patients from 1975 to 2011. The actual 5-year survival for all stages in 1975 was 0.9% and rose to 4.2% in 2011 (p < 0.001). Surgically resected patients saw an increase in actual 5-year survival from 1.5% in 1975 to 17.4% in 2011 (p < 0.001). Some 0.8% of non-resected patients passed the 5-year mark in 1975 while 0.9% did so in 2011 (p = 0.121).

## Discussion

To our knowledge, this study represents the largest evaluation of LTS in pancreatic ductal adenocarcinoma in the modern era. As stated earlier, most previous studies of actual survival have been single centre reports with a limited number of LTS for analysis^6–8,13–24^. A few population-based studies of survival trends in pancreatic cancer have been published^25–28^. Still, most of these studies make use of older data and do not investigate actual survivors of the disease further. Kardosh et al^27^ was the only population-based study investigating predictors of actual survival, including 39,460 patients in the California Cancer Registry from 1988 to 2009. However, this study evaluated prognostic factors in a timeframe overlapping the pre- and post-ESPAC-1-era (2004–present). To achieve comparability as well as generalisability of our findings, we set out to analyse the largest recent dataset of actual LTS.

In this study, we exclusively evaluated patients with microscopically confirmed pancreatic adenocarcinoma in order to provide reliable survival data. We found lower age, female gender, histologic grade, tumour size, T-stage, M-stage, surgical resection and receipt of chemotherapy to be independent prognostic factors of long-term survival. In surgically resected patients, nodal status was also an independent factor. Survival plots demonstrated a marked increase in actual survival for surgically resected patients, from 1.5% in 1975 to 17.4% in 2011. Patients of all stages showed an actual survival of just 4.2% in 2011, and actual survival without surgical treatment remained below the 1% mark, showing no improving trend since the start of data collection.

Statistically determined factors may not completely predict the patients that eventually achieve long-term survival^14^. In our cohort, disparities between actual patient characteristics and the prognostic modelling were found. While the LTS generally had more favourable clinicopathological features, the presence of aggressive characteristics (e.g. poor/anaplastic differentiation, late stage disease) or lack of surgical resection did not preclude long-term survival. The presence of a high number of patients with favourable prognostic factors in the LTS cohorts is believed to indicate inherent differences in biological tumour behavior^14,18,24,27^. Attempts have been made at deciphering this difference through molecular studies. Molin et al^29^ conducted whole-exome sequencing of patients who survived ≥ 10 years. KRAS mutations were identified in 94% from LTS, while TP53, SMAD4, and CDKN2A mutations were found in 69%, 26%, and 17%, respectively. RNF43 mutations were identified in 11%. Their data could not demonstrate any difference in somatic mutations in carcinomas from LTS compared to available data from unselected pancreatic cancer patients. In another study, Balachandran et al^30^ conducted extensive immunoprofiling of patients with long-term survival (overall survival > 3 years from surgery; median survival 6 years) and patients with short-term survival (overall survival < 1 year from surgery; median survival 0.8 years). They found that long-term survivors harbour neoantigens with unique qualities as T-cell targets in pancreatic cancer and propose a role for immunotherapy in pancreatic cancer based on directed neoantigen targeting.

As expected, stage and surgery had the strongest predictive capabilities among all our measured variables. Major improvements have been made in the surgical management of pancreatic cancer over the past decades due to advances in surgical technique and perioperative care. Operative mortality associated with pancreatoduodenectomy has decreased from around 25% in the 1970s to under 2% at high-volume centres in recent series, and the focus has now shifted from surviving the operation to surviving the cancer^31^. Importantly, the introduction of chemotherapy has come to greatly improve median survival and actuarial 5-year survival rates following surgical resection^12^. However, the impact of chemotherapy on actual LTS remains less clear. We found that chemotherapy was independently associated with LTS. Several single-centre studies have reported actual 5-year survival among resected patients in the range of 8.1–30.7%^6–8,13–24^, which is in line with the current study, but none of these studies could statistically confirm the independent clinical benefit of chemotherapy for LTS as shown by us. Furthermore, the relatively stable proportion of localized disease over time and the dismal long-term survival rate (4.2%) for the whole cohort in our study underscore the urgent need for improvements in diagnostic procedures alongside improvement in oncological therapy. A predominant proportion of patients progress asymptomatically, which calls for efforts to bring about early detection of sporadic pancreatic cancer. It has been proposed that a strategy involving an increase in the percentage of individuals diagnosed with IA cancer could, with the current treatment arsenal, greatly improve survival and the chance for cure^2^. As we demonstrated in our cohort, the greatest reduction in actual survival exists between AJCC stages IA and IB. The development of effective early detection programs has been conceptualized^32^ and involves the identification of novel and existing biomarkers of pancreatic cancer for use in a high-risk cohort. Such development requires strategic collaboration between academia, industry and government.

This study has several limitations inherent to the use of a multi-institutional registry. The SEER registry conducts quality control activities regularly to ensure data accuracy and consistency. However, the SEER registry may underestimate treatments, such as chemotherapy and radiotherapy, due to outpatient treatment, or patients leaving the registry catchment area for treatments^33^. We included only microscopically confirmed cases, a criterion that improves reliability of data, but may have contributed to the high resection rate (18.5%) observed in our patient material. Furthermore, the registry also contains missing values. We handled missing values by multiple imputation technique, which is a suitable method that reduces selection bias and improves generalisability, but necessitates caution when interpreting the results.

In conclusion, our study identified predictive factors for actual long-term survival in pancreatic ductal adenocarcinoma using population-based real-world data. Actual survival has only marginally improved over the past decades and patients of all stages still retain a 5-year survival below 5%. The greatest reduction of long-term survival is observed from stages IA to IB. As the proportion of patients with localized stage disease has remained exceedingly low over time, this calls for further strategic developments of early detection tools. Furthermore, given the poor survival rate even after potentially curative surgery, novel oncological treatments need to be developed to address occult, systemic, micrometastatic disease. The future therapeutic developments of pancreatic cancer may be aided by better molecular understanding of the disease as a whole, but also by increased knowledge obtained by studying LTS.

## Methods

### Study population and selection criteria

The SEER database is a population-based cancer registry that assembles data related to demographics, incidence and survival of cancer patients in the United States. Data were obtained from all cancer registries participating in the SEER program using SEER*Stat version 8.3.6 (November 2018 data submission). The study was approved by the Ethics Committee for Clinical Research at Lund University (Ref 2016/100) and conducted in accordance with the STROBE guidelines^34^.

All patients with pancreatic cancer registered in the SEER database between 1975 and 2011 were included in the study group. Patients were identified on the basis of the International Classification of Diseases for Oncology, third edition (ICD-O-3) for tumours of the exocrine pancreas: C25.0, C25.1, C25.2, C25.3, C25.7, C25.8 and C25.9. Only cases with microscopically confirmed infiltrating pancreatic ductal adenocarcinoma (ICD-O-3 histology codes 8140/3 and 8500/3 respectively) were selected. Patients with missing values on duration of follow-up or cause of death were excluded. The SEER historic stage A was used in the description of the overall population in order to present a uniform classification, as the American Joint Committee on Cancer (AJCC) staging system was not available for older time periods. The latest date of follow-up was on December 31, 2016. Patients deceased from non-cancerous causes within 5 years of diagnosis were excluded.

Information was available on age, gender, calendar year of diagnosis, tumour location, histological grade, tumour size, stage (SEER historic tumour stage A and AJCC/TNM staging), surgical resection, chemotherapy, radiotherapy, survival time and vital status.

The primary endpoint was actual 5-year survival.

### Statistical analysis

We compared clinicopathological variables between long-term survivors (LTS; actual survival ≥ 5 years) and short-term survivors (STS; actual survival < 5 years) using Mann Whitney U test for continuous variables and Pearson’s Chi-square (χ^2^) test for categorical variables. A trend analysis of actual 5-year survival by year in surgically resected, non-resected, and all patient stages was conducted. Trend curves were tested for significance using linear-by-linear association. Factors associated with actual 5-year survival were assessed with logistic regression. Any variable from univariable logistic regression with a p value < 0.25 was selected as a candidate for the multivariable analysis. In the iterative process of variable selection, covariates were removed from the model if they were nonsignificant and not a confounder, as described by Hosmer–Lemeshow^35^, resulting in a main effect model. Missing values were imputed using the multiple imputation with chained equations technique, as described by White and colleagues^36^. The imputation method was predictive mean matching. The number of iterations for each chain was ten, as was the number of imputed data sets. Statistical analyses were performed using IBM SPSS version 26 and Stata/MP 14.2.

### Informed consent

The SEER registry contains strictly de-identified patient data. The Ethics Committee for Clinical Research at Lund University (Ref 2016/100), Sweden, approved the study protocol and waived the need for written informed consent from the participants.

## Data Availability

The datasets generated during and/or analysed during the current study are available from the corresponding author on request.
